# Stability-indicating spectrophotometric manipulations for the determination of Letrozole in the presence of its alkali-induced degradation products; towards whiteness and ChlorTox scale perspectives

**DOI:** 10.1186/s13065-025-01416-2

**Published:** 2025-03-08

**Authors:** Nourhan A. Abd El-Fatah, Manal Mohammed Fouad, Maha A. Hegazy, Ghada M. El-Sayed

**Affiliations:** 1 Analytical Chemistry Department, Faculty of Pharmacy, October University for Modern Sciences and Arts (MSA), 6th October City, Giza, 11787 Egypt; 2 Analytical Chemistry Department, Faculty of Pharmacy, Al-Azhar University, Nasr City, Cairo 11651 Egypt; 3 Analytical Chemistry Department, Faculty of Pharmacy, Cairo University, Kasr El-Aini Street, Cairo, 11562 Egypt

**Keywords:** Letrozole, Alkali-induced degradation products, Spectrophotometry, Whiteness evaluation, ClorTox assessment

## Abstract

Letrozole (LTZ) is an established first hormonal treatment for breast cancer, yet it was found to be highly susceptible for degradation in alkaline medium due to the presence of cyano phenyl group. Consequently, three stability-indicating spectrophotometric manipulations for LTZ quantification in presence of its alkali-induced degradation products were developed for the first time; ensuring methods’ simplicity, sensitivity and accuracy. The first method was *Second derivative (D*_*2*_*)* by recording peak amplitude of the drug at 226.8 nm. The second method was *Ratio difference (RD)* where the peak amplitude were recorded at wavelengths 240.0 nm and 258.0 nm. The third method was *First derivative of ratio spectra (DD*_*1*_*)* through recording peak amplitude at 246.0 nm. Linearity ranged from 1.00 to 16.00 µg/mL for D_2_, while from 3.00 to 16.00 µg/mL for RD and DD_1_ with adequate recoveries 100.02 ± 1.371, 100.05 ± 1.972 and 100.40 ± 1.223 for D_2_, RD and DD_1_, respectively. The proposed methods were validated as per ICH guidelines, and were successfully applied for determination of LTZ in bulk powder, laboratory-prepared mixtures, and pharmaceutical formulation. The Whiteness tool, using the RBG12 algorithm, was employed to evaluate environmental aspects, as well, ChlorTox scale was used to assess chemicals’ hazards for this study.

**Supplementary Information:**

The online version contains supplementary material available at 10.1186/s13065-025-01416-2.

## Introduction

**Fig. 1:**
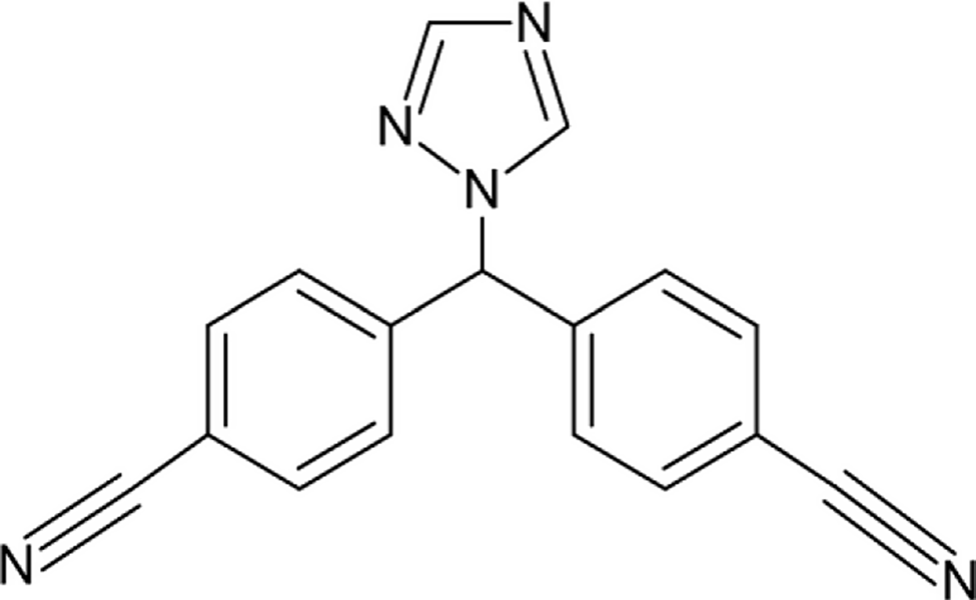
Chemical structure of Letrozole

Letrozole (LTZ) (Fig. 1); named chemically as [4,4-(1 H-1,2,4-Triazole-1-yl methylene) bis-benzonitrile)] [1] is a third-generation aromatase inhibitor drug that plays a vital role in breast cancer due to its proven efficacy as a first-line hormonal therapy versus metastatic and non-metastatic cases [2]. It reduces the circulating estrogen in plasma levels by selective inhibition of testosterone conversion into estradiol [3]. Literature revealed quantitative methods reported for determination of LTZ such as: spectrophotometric [4–6], chromatographic [7–19], and electrochemical [20–25].

In advanced analytical laboratories, stability-indicating studies expose significant need to be developed for confirming drug stability and its regulatory necessity [26]. A stability-indicating method is considered an ideal one when it determines the drug and resolve its degradation products [27]. Additionally, spectrophotometric methods-based efficiency returns to its simplicity, fastness, accuracy, economical, validity, applicability, besides, majority of compounds exhibit absorbance in the UV-region [26].

As reviewed in the reported chromatographic literature, LTZ was found to be more labile for alkaline degradation, yet it showed stability towards other degradation routes and this may be attributed to the presence of cyano phenyl group in LTZ [7, 9]. However, there is no reported spectrophotometric methods concerned with the determination of LTZ in the presence of its degradation products. Accordingly, in this research, three spectrophotometric methods were manipulated for resolving the overlapping between LTZ and its alkali-induced degradation products in bulk powder or pharmaceutical formulation. The developed methods are *Second derivative (D*_*2*_*)*, *Ratio difference (RD)*, and *First derivative of ratio spectra (DD*_*1*_*)*. Besides, this work has been assessed regarding sustainability and eco-friendliness by white analytical chemistry (WAC) [28] and unified greenness theory (UGT) [29] perspectives; through the aid of **RGB12 algorithm** and **ChlorTox Scale** tools, respectively.

## Experimental

### Instrumentation

All absorbance measurements were carried out using Shimadzu UV-Vis 1601 double beam spectrophotometer, with two matched 1.00 cm path-length quartz cells (Tokyo, Japan). Scanning was performed at 200.0–400.0 nm range, with 0.1 nm intervals, and spectra were obtained using the UV-Probe (version 2.10) system software. Electronic balance (Shimadzu, Japan), ultrasonic water bath (Elma, Germany), and pH meter (Jenway 3505, UK) were used for solutions’ preparations. TLC plates-precoated with silica gel F_254_, 0.25 mm thickness (Merck, Darmstadt, Germany) was also used.

### Materials and reagents

Letrozole (LTZ) reference standard was purchased from Sigma Aldrich (Missouri, USA), and its purity was found to be 100.17% ± 0.789 as per reported spectrophotometric method [5]. All chemicals used in this study were of analytical grade, while all solvents were of spectroscopic grade. Hydrochloric acid (HCl), sodium hydroxide (NaOH), and hexane (melting point: 60–80 ^o^C) were purchased from El-Nasr Pharmaceutical Company (Cairo, Egypt). Absolute ethanol was obtained from PioChem Laboratory Chemicals (Cairo, Egypt). Ethyl acetate was bought from Advent ChemBio Pvt. Ltd. (Rabale, India). The pharmaceutical dosage form; Femara^®^ tablets (Batch no. **SAWJ9**; Novartis Pharmaceutical Company, Egypt**)** labeled to contain 2.5 mg of LTZ per tablet, was purchased from the Egyptian market.

### Solutions

#### **Letrozole stock and working standard solutions**

Letrozole stock standard solution (1000.00 µg/mL) was prepared by transferring 1.0 × 10^6^ µg of LTZ into 100-mL volumetric flask and the volume was completed with ethanol. Ten mL of this solution were transferred to a 100-mL volumetric flask and diluted to volume with the same solvent to obtain a working solution of 100.00 µg/mL.

#### Alkali-induced degradation products solution

Alkali-induced degradation of LTZ was prepared as per the reported conditions [9]. An appropriate volume (10.0 mL) of 2.0 N NaOH was added to 5.0 mL of LTZ stock were refluxed at 75 °C for 0.5 h. After that, solution was cooled, neutralized by 2.0 N HCl, transferred to 50-mL volumetric flask and completed to volume with ethanol to obtain a solution estimated to contain alkali-induced degradation products equivalent to 100.00 µg/mL. This procedure was carried out in dark to prevent photo-degradation.

Compete degradation of LTZ was confirmed using TLC development system composed of hexane: ethyl acetate (1:1 v/v). Consequently, FTIR spectroscopy was used for confirming the degradation products and verifying the reported degradation mechanism [9].

### Procedures

#### Construction of calibration curves

##### Spectral characteristics

Zero-order spectra (D_0_) of prepared LTZ solutions having concentrations ranging from 1.00 to 16.00 µg/mL were scanned at 200.0–400.0 nm range with 0.1 nm intervals, against ethanol as a blank. Moreover, the spectrum of 10.00 µg/mL of the alkaline induced degradation products was recorded at the same wavelength interval. The obtained data were stored in the computer, in which different manipulation steps were further applied to the stored data.

##### Zero-order spectrophotometric method (D_0_)

Zero-order spectrophotometric method (D_0_) of LTZ prepared solutions (1.00–16.00 µg/mL) were recorded at 200.0–400.0 nm range with 0.1 nm intervals, against ethanol as a blank, and data was stored on the computer. Quantitative determinations of LTZ alone were carried out at maximum wavelength (λ_max_) 239.0 nm. A calibration graph was plotted using LTZ absorbance versus its corresponding concentrations, computing its regression equation.

##### Second derivative (D_2_) spectrophotometric method

Second derivative of the recorded zero-order spectra of LTZ and its alkali-induced degradation products were computed using delta lambda (Δλ) = 8 nm and scaling factor 100. The obtained peak amplitudes of D_2_ spectra were measured at 226.8 nm. A calibration graph was constructed using LTZ peak amplitude versus its corresponding concentrations, followed by calculating the corresponding regression equation.

##### Ratio difference (RD) spectrophotometric method

Zero-order spectra (D_0_) of LTZ were divided by the spectrum of 10.00 µg/mL alkali-induced degradation products; obtaining ratio spectra to be stored on the computer. Targeted amplitudes of those ratio spectra were measured at 240.0 and 258.0 nm. A calibration graph correlating amplitude difference of ratio spectra (*ΔP*_240.0– 258.0_) to LTZ corresponding concentrations was plotted, computing its regression equation.

##### First derivative of ratio spectra (DD_1_) spectrophotometric method

Zero-order spectra (D_0_) of LTZ were divided by the spectrum of 10.00 µg/mL alkali-induced degradation products; obtaining ratio spectra to be stored on the computer. Afterwards, further manipulation using the first derivative (Δλ = 8 nm and scaling factor 10) for those stored ratio spectra (DD_1_) was performed and recorded. A calibration graph correlating peak amplitude of DD_1_ at 246.0 nm to LTZ corresponding concentrations was plotted, computing its regression equation.

### Methods’ validation

#### Linearity

In a set of 10-mL volumetric flasks, aliquots for D_2_ manipulation (0.10–1.60 mL), as well other aliquots for RD and DD_1_ manipulations (0.30–1.60 mL) of LTZ working standard solution (100.00 µg/mL) were accurately transferred. Volumes were completed with ethanol, to obtain solutions of concentration 1.00–16.00 µg/mL for D_2_, while 3.00–16.00 µg/mL for RD and DD_1_, scanned over 200–400 nm wavelength range. Calibration graphs for the three proposed methods were plotted using LTZ response (absorbance/ peak amplitude) versus its corresponding concentrations and regression equations were calculated.

#### Accuracy

Methods’ accuracy were evaluated separately by mean recovery % of three different LTZ concentrations (3.00, 7.00, 14.00 µg/mL) in triplicate from the corresponding regression equation of each developed method.

#### LOD and LOQ

Limit of detection (LOD) and limit of quantitation (LOQ) were calculated for the three proposed methods as follows: **LOD = 3.3 x**$\:\frac{\mathbf{S}\mathbf{D}\:}{\mathbf{S}}$ and **LOQ = 10 x**$\:\frac{\mathbf{S}\mathbf{D}}{\mathbf{S}}$, where **SD** is the standard deviation of y-intercept, and **S** is the slope.

#### Precision

Intra-day precision (within the same day) and inter-day precision (on three consecutive days) were assayed through pure LTZ analysis at three different concentration levels (3.00, 7.00, 14.00 µg/mL) in triplicate. Their corresponding RSD% (relative standard deviation) was adequately calculated.

#### Robustness

Robustness of the proposed methods was assessed through slight changes in spectral measurements (± 2 nm) that may arise within the spectrophotometric analysis.

#### Specificity

Methods’ specificity were evaluated by the analysis of LTZ in laboratory-prepared mixtures, containing both the intact drug and its alkali-induced degradation products.

### Application to laboratory-prepared mixtures

Laboratory-prepared mixtures containing different ratios of LTZ and its alkali-induced degradation products were organized from their corresponding working solutions (100.00 µg/mL) as per the previously mentioned procedure. Their spectra were recorded from 200.0 to 400.0 nm and data was stored on the computer. LTZ concentrations were calculated using the corresponding regression equation for each proposed method.

### Application to pharmaceutical formulation

Ten tablets of Femara^®^ were accurately weighed and finely grinded. An equivalent amount of powder to 2.5 mg of LTZ was accurately weighed and transferred into a 25-mL volumetric flask, then volume was completed with ethanol, sonicated for 15 min, to obtain a final concentration of 100.00 µg/mL that is further filtered using a 0.45 μm membrane filter. Subsequent procedures were performed as illustrated *underSect.* (Linearity). The recovery of the proposed methods was further assessed by applying the standard addition technique.

## Results and discussion

### LTZ alkali-induced degradation products

According to the literature, two stability studies described all degradation routes (alkaline, acidic, thermal, photolytic, and oxidative) of LTZ were reported [7, 9]. Those studies confirmed that LTZ is more labile to alkaline degradation, yet it showed considerable stability towards other conditions. This may be attributed to the presence of cyano phenyl group in LTZ chemical structure which undergoes to either partial hydrolysis (amide degradation product), or further hydrolysis (carboxylic acid degradation product).

**Fig. 2:**
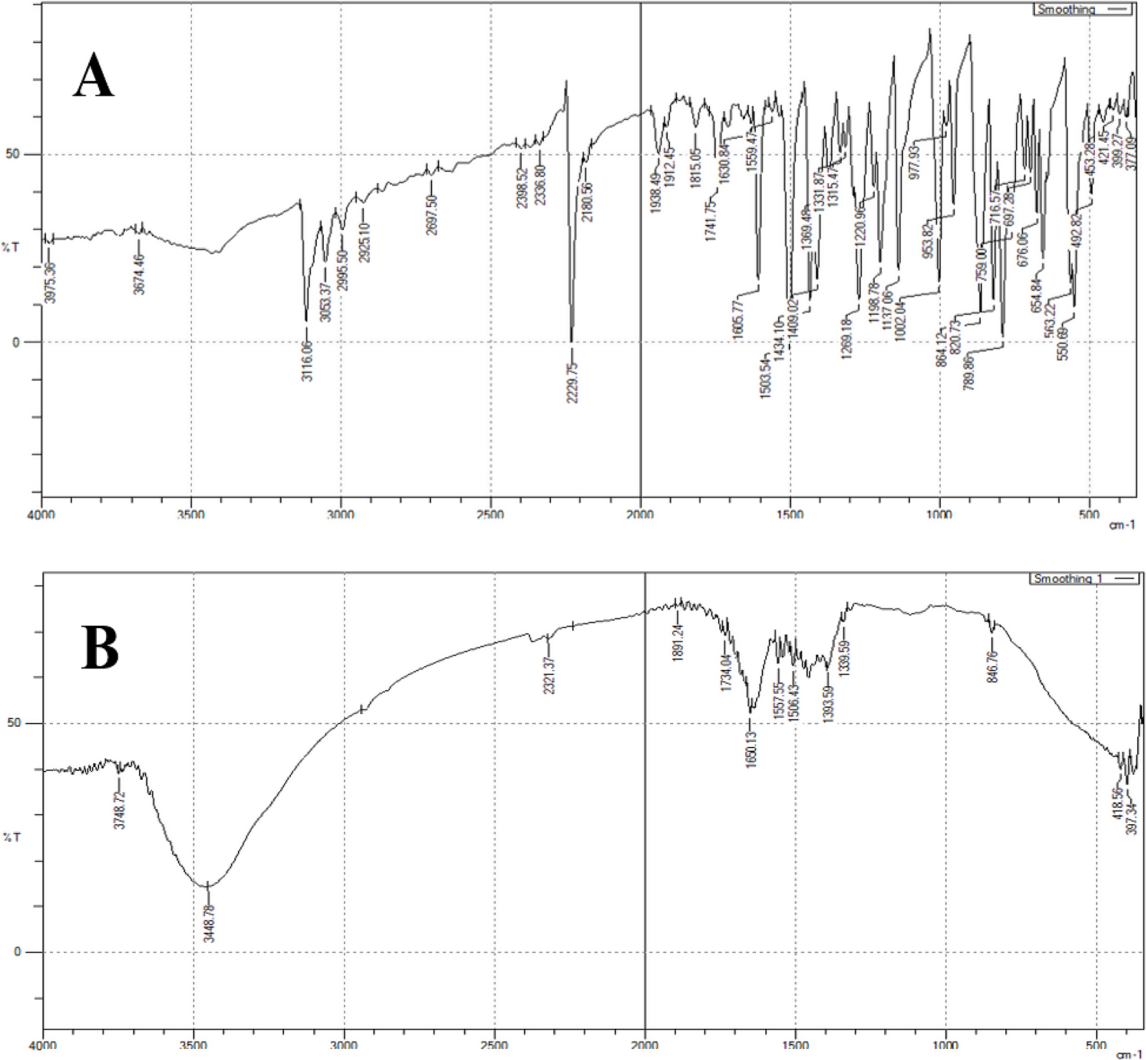
IR spectrum of (**A**) Letrozole, and (**B**) alkali-induced degradation products

**Fig. 3:**
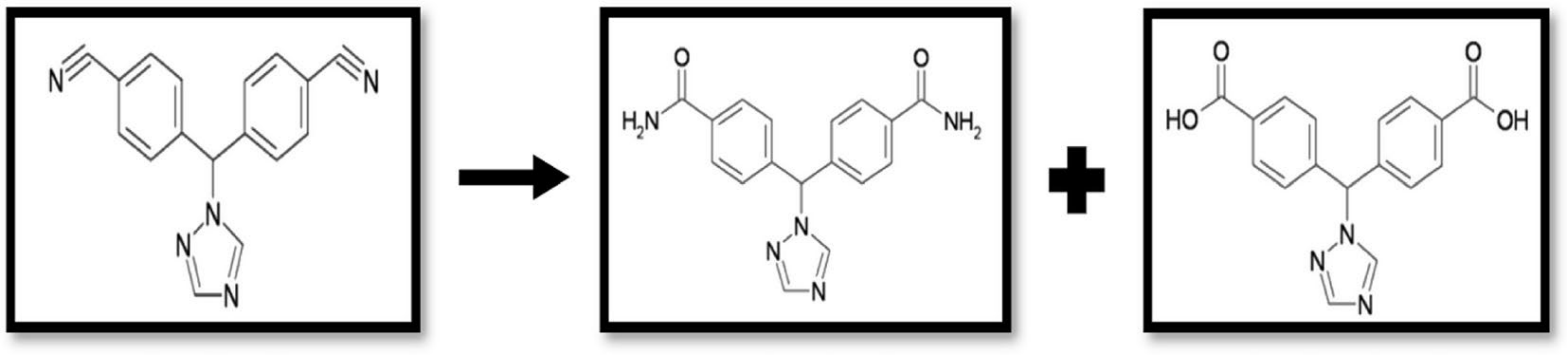
Suggested alkali-induced degradation pathway of Letrozole

Letrozole was completely degraded as indicated through the changed R_f_ value of TLC; where the intact drug exhibits R_f_= 0.40, and the two degradation products; one which is the amide degradation product with R_f_= 0.27, while the second is the acid degradation product having R_f_= 0.14 (Fig. S1). Moreover, FTIR spectroscopic investigation (Fig. 2) was utilized for confirming the suggested alkali-induced degradation pathway (Fig. 3). It ensures the disappearance of cyano phenyl groups’ characteristic peak at nearly 2200 cm^− 1^ in LTZ intact, whereas the appearance of carbonyl group peak for amide product at 1650 cm^− 1^, additionally the broad band from 2500 to 3500 cm^− 1^ confirms the presence of hydroxyl group of the carboxyl group, which mask the forked peak of the NH_2_ of the amide degradation product.

### Spectrophotometric methods

#### Zero-order spectrophotometric method (D_0_)

**Fig. 4:**
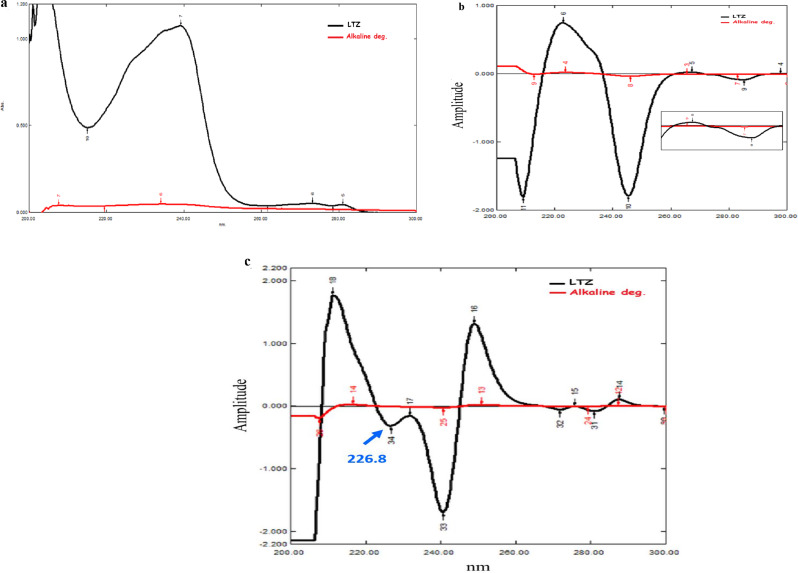
(**a**) ***Zero-order*** UV spectra of Letrozole (10.00 µg/mL) with its alkali-induced degradation products (10.00 µg/mL) against ethanol solvent as a blank, **overlapped** in a range between 200–300 nm. (**b**) ***First derivative*** UV spectra of Letrozole (10.00 µg/mL) with its alkali-induced degradation products (10.00 µg/mL) against ethanol solvent as a blank, **with no zero-crossing** in a range between 200–300 nm. (**c**) ***Second derivative*** UV spectra of Letrozole (10.00 µg/mL) with its alkali-induced degradation products (10.00 µg/mL) against ethanol solvent as a blank, **with zero-crossing at*226.8 nm*** in a range between 200 and 300 nm.

Zero-order spectrophotometric method of LTZ and its alkali-induced degradation products (10.00 µg/mL) showed an overlapping in the range of 200.0–290.0 nm (Fig. 4a), preventing the direct spectrophotometric measurements of the drug in presence of its degradation products. Probably, it could be considered a quantitative method for determination of LTZ alone in bulk or pharmaceutical formulation over the concentration range 1.00–16.00 µg/mL using its corresponding regression equation (Fig. S2). Consequently, this method revealed superiority over other reported spectrophotometric methods for LTZ determination. This returns to ethanol usage that supports method greenness and also to the higher sensitivity of our proposed method. However, for the reported literature, methanol was used instead [5] with lesser sensitivity (2.00–20.00 µg/ml) [4], or otherwise, ethanol was used with narrower linearity range (1.00–10.00 µg/ml) [6].

#### Second derivative (D_2_)

Derivative spectrophotometric is considered a common analytical method, as it is used for extracting qualitative and quantitative data from any spectra composed of unresolved bands, and for eliminating baseline shifts/tilts effects [27]. After the failure of first derivative (D_1_) manipulation to resolve this simultaneous determination, for the disappearance of any zero-crossing as shown in Fig. 4b, D_2_ method was developed based on measuring the peak amplitude of D_2_ spectrum of LTZ selectively at 226.8 nm that corresponds to zero-crossing of its alkali-induced degradation products (Fig. 4c). Regarding D_2_ method optimization and good spectral resolution, the most effective Δλ was found to be 8 nm with scaling factor equals 100. This showed an appropriate signal to noise ratio. Accordingly, LTZ can be quantitatively determined over the concentration range 1.00–16.00 µg/mL using its corresponding regression equation (Fig. S3a).

#### Ratio difference (RD)

Ratio difference depends on eliminating the interfering component (alkali-induced degradation products), where the difference at any two points of this interferent will always be zero. The method comprises two critical steps, the first is the choice of the divisor; the selected divisor should compromise between minimal noise and maximum sensitivity. The divisor concentration of 10.00 µg/mL gave the best results. The second critical step is the choice of the wavelengths at which measurements are recorded. Any two wavelengths can be chosen provided that they exhibit different amplitudes in the ratio spectrum and give good linearity at each wavelength individually; best results were obtained at 240.0 and 258.0 nm (ΔP 240.0–258.0 nm). RD is considered a prominent method in resolving the strictly overlapped spectra without the need of prior separation; due to its easiness, replicability, accuracy, and reduced manipulating steps [30].

**Fig. 5:**
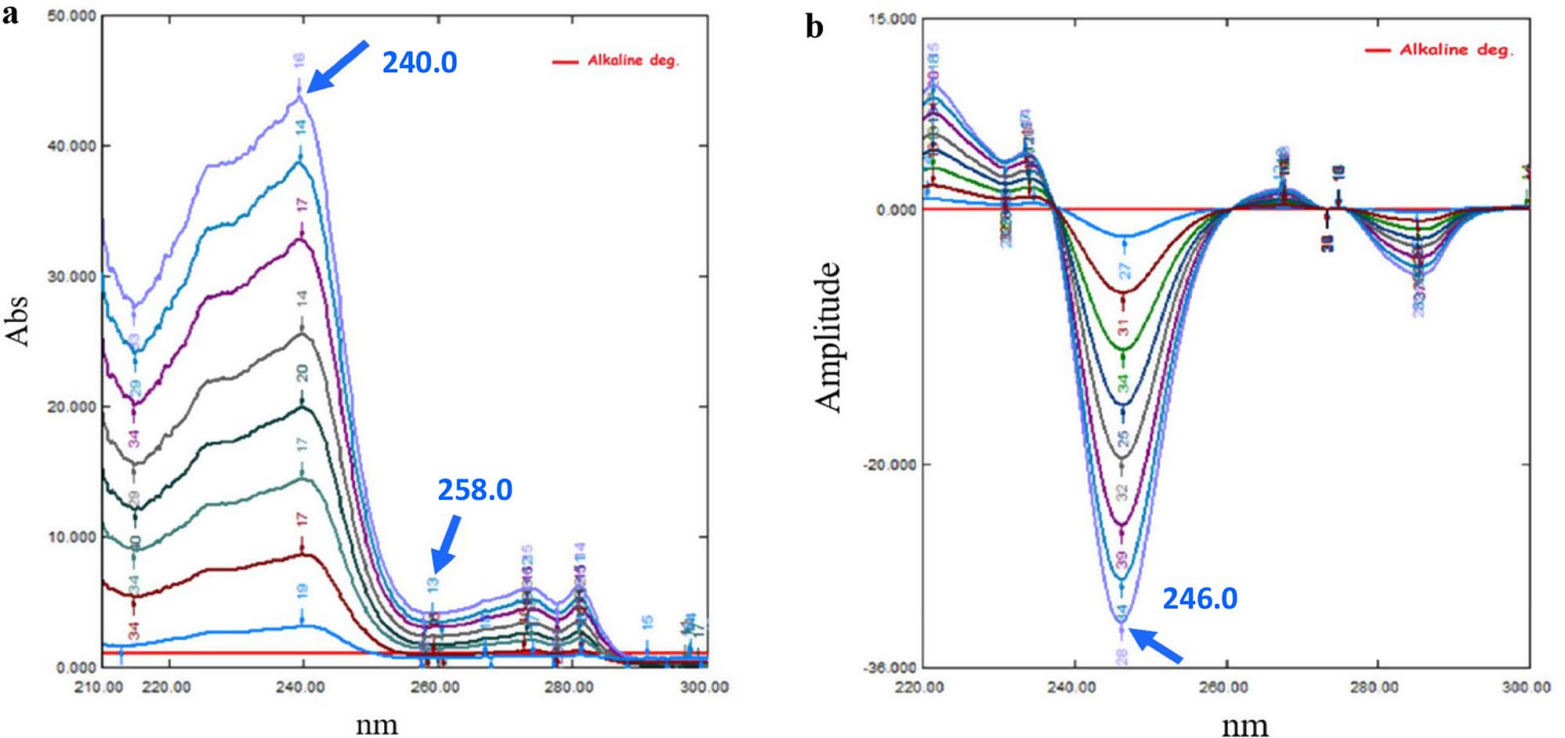
Linearity of Letrozole by (5a) Ratio spectra (3.00–16.00 µg/mL) and (5b) First derivative of ratio spectra (3.00–16.00 µg/mL) using the spectrum of alkali-induced degradation products (10.00 µg/mL) as a divisor

Ratio spectra were obtained through dividing the LTZ concentrations spectra (3.00–16.00 µg/mL) by the spectrum of the alkali-induced degradation products (10.00 µg/mL) as a divisor, then measuring the difference in amplitude (ΔP) between targeted wavelengths (240.0 and 258.0 nm), through subtracting the two amplitude values at those wavelengths (Fig. 5a). ΔP is directly proportional to LTZ concentrations; independent to the interfering component. LTZ concentration was calculated using the linear regression equation represented in Fig. S3b.

#### First derivative ratio spectra (DD_1_)

The principle of DD_1_ depends mainly on cancelling the whole spectrum of the alkali-induced degradation products (interfering substance), in addition, there is no critical need for selecting a specific wavelength used for calibration as in D_2_ [27]. DD_1_ is figured out mainly by obtaining ratio spectra as mentioned *underSect.* (Ratio difference (RD)), then manipulating first derivative (Δλ = 8 nm and scaling factor 10) on this ratio spectra. Linear LTZ concentrations (3.00–16.00 µg/mL) (Fig. 5b) were calculated at 246.0 nm amplitude using its corresponding regression equation as represented in Fig. S3c. DD_1_ has essential advantages, including its enhanced sensitivity, selectivity, improved spectral resolution, reduced baseline drift, and minimized interference in a mixture [30].

### Methods’ validation

According to the International Conference on Harmonization (ICH) Q2 guidelines [31], validation of the proposed three spectrophotometric methods was performed.

#### Linearity

Linearity range of LTZ was found to be 1.00–16.00 µg/mL for D_2_ method, while 3.00–16.00 µg/mL for RD and DD_1_ methods, obtaining acceptable correlation coefficient **(r)** values; 0.9991, 0.9995, 0.9994 approaching unity for D_2_, RD, and DD_1_, respectively as represented in Table 1.

#### Accuracy

As revealed in Table 1, methods’ accuracy were found to be within the acceptable range: 100.02 ± 1.371, 100.05 ± 1.972 and 100.40 ± 1.223 for D_2_, RD and DD_1_, respectively.

#### LOD and LOQ

Ratio difference method showed the least LOD (0.292 µg/mL) and LOQ (0.885 µg/mL); ensuring maximum sensitivity among other methods. Other results were signified in Table 1.

#### Precision

Precision of each method was adequately calculated from their corresponding RSD% values (< 2%); ensuring precise methods as shown in Table 1.

#### Robustness

Table 1. confirmed each calculated RSD% values (< 2%) for the robustness of three proposed results.

**Table 1:** Regression and validation parameters of the proposed spectrophotometric methods for Letrozole determination

Parameters		D_2_	RD	DD_1_
Wavelength (nm)		226.8	(*ΔP*_240.0– 258.0_)	246.0
Linearity range (µg/mL)		1.00–16.00	3.00–16.00	
**Regression parameters**				
Slope		0.0393	2.4193	2.0138
Intercept		− 0.0038	0.7450	0.6093
SE		0.471	0.407	0.385
Correlation coefficient (r^2^)		0.9991	0.9995	0.9994
LOD (µg/mL)		0.379	0.292	0.345
LOQ (µg/mL)		1.148	0.885	1.045
Accuracy* (mean ± SD)		100.02 ± 1.371	100.05 ± 1.972	100.40 ± 1.223
Inter-day precision (RSD %) **				
Conc. (µg/mL)	3.00	0.457	0.351	0.242
	7.00	0.682	0.289	0.564
	14.00	0.232	0.411	0.327
Intra-day precision (RSD %) **				
Conc. (µg/mL)	3.00	0.399	0.317	0.141
	7.00	0.502	0.458	0.301
	14.00	0.416	0.252	0.611
Robustness (RSD %) **		0.282	0.441	0.522

* Average of three determinations

** Average Relative standard deviation of three triplicate concentrations

#### Specificity

The three proposed methods exposed satisfactory results regarding specificity of the laboratory-prepared mixtures. The obtained mean recoveries were 100.89 ± 0.389, 100.36 ± 1.361, and 100.49 ± 1.122 for D_2_, RD, and DD_1_, respectively (Table 2).

### Application to laboratory-prepared mixtures

Table 2 revealed the laboratory-prepared mixtures of LTZ and its alkali-induced degradation products in different ratios within the anticipated linearity; each mixture was separately analyzed using the three proposed methods. The recoveries obtained for each method proved that LTZ can be quantified without the interference of its alkali-induced degradation products up to 90% of its degradation products; confirming high specificity levels of the developed methods.

**Table 2:** Laboratory-prepared mixtures of Letrozole in presence of its alkali-induced degradation products using the proposed spectrophotometric methods

Ratio %	* Recovery %		
LTZ: Alkali-induced degradation products	D_2_	RD	DD_1_
9: 1	100.88	98.44	101.56
8: 2	100.45	99.02	100.55
7: 3	101.34	101.62	101.98
6: 4	101.27	101.80	99.81
5: 5	101.17	101.41	101.09
4: 6	101.02	100.61	98.38
3: 7	100.76	101.10	100.47
2: 8	100.25	98.91	100.10
1: 9	91.09 **	89.41 **	90.21 **
Recovery (Mean % ± SD)	100.89 ± 0.389	100.36 ± 1.361	100.49 ± 1.122

* Average of three determinations

** Rejected values

### Application to pharmaceutical formulation

The proposed methods were successfully applied for the quantitative determination of LTZ in Femara^®^ tablets. The obtained results were found to be within the acceptance limit of the labelled claim, ensuring that excipients have no effect on the analysis. Also, methods’ validity were proven using the standard addition technique as demonstrated in Table 3.

**Table 3:** Quantitative determination of Letrozole in Femara^®^ tablets by the proposed spectrophotometric methods and application of standard addition technique

Pharmaceutical formulation Femara^®^ (2.5 mg/ tablet)	* Found % ± SD					
	D_2_		RD		DD_1_	
Batch no. **SAWJ9**	99.84 ± 1.012		99.55 ± 0.911		99.73 ± 0.752	
**Standard addition technique**	**D**_**2**_		**RD**		**DD**_**1**_	
**Added (µg/mL)**	**Found**	**Recovery %**	**Found**	**Recovery %**	**Found**	**Recovery %**
2.00	1.99	99.50	2.03	101.5	1.99	99.5
4.00	4.02	99.25	4.03	100.75	4.13	99.75
6.00	6.04	99.83	5.98	99.67	6.16	101.67
**Recovery (Mean % ± SD)**	100.22 ± 0.632		100.64 ± 0.920		100.31 ± 1.187	

* Average of three determinations

### Statistical analysis

Results obtained by the proposed methods were compared with other reported method [5], revealed that calculated *Student’s T-tests* and *F-tests* didn’t exceed the tabulated values, ensuring insignificant difference between methods’ accuracy and precision (Table 4). Also, One-way ANOVA test was used for comparing the three proposed spectrophotometric methods; obtaining satisfactory results that exposed no significant variations among them; as the calculated F-values didn’t exceed the critical one (Table 5).

**Table 4:** Statistical analysis for the obtained results from the proposed methods and the reference method

Statistical Term	D_2_	RD	DD_1_	Reference method ^a^
Mean %	99.79	100.75	100.54	100.17
SD^b^	1.172	1.152	1.089	0.789
n	8	8	8	8
Variance	1.373	1.327	1.187	0.623
*Student’s t-test* (2.145)^c^	0.761	1.175	0.778	-
*F-test* (3.787)^c^	2.203	2.128	1.904	-

^a^ Reported spectrophotometric method

^b^ Standard Deviation

^c^ Values in parentheses are the critical t- and F-values at *P* = 0.05

**Table 5:** One-way ANOVA results for Letrozole determination using the proposed spectrophotometric methods

Source of variation	Sum of squares	Degree of freedom	Mean of squares	F-value^a^	*P*-value^a^	Critical F^a^
*Between columns*	4.297	3	1.432	1.270	0.304	2.946
*Within columns*	31.570	28	1.127			
*Total*	35.866	31				

^a^ No significant difference between the proposed methods using single factor ANOVA at *P* < 0.05

### Methods’ assessment

In 2021, a newly sustainability evaluating principle named White Analytical Chemistry (WAC) was released, serving additional aspects rather than the known twelve GAC principles such as economical cost, method validity, and method efficiency. This was achieved using **RGB12 algorithm** tool, where it includes three main attributes defined by three categorized colors [Red (R), Green (G) and Blue (B)], each category is subdivided into other four parameters named [(R1, R2, R3, R4), (G1, G2, G3, G4) and (B1, B2, B3, B4)], relevant to analytical performance, ecological impact, and commercial measures respectively. Accordingly, white is the net color produced from mixing the three colors together. Scoring for each attribute ranges from 0 to 100, where 0 is the worst score, while 100 is the appropriate one as represented in [28]. The proposed methods *D*_*2*_, *RD*, and *DD*_*1*_ showed perfect whiteness scoring as follows: 91.5, 89.9, and 88.6 respectively; revealing the balance between the mentioned attributes, as well, maintaining sustainability goals.

Recently in 2023, Chloroform-oriented toxicity is a reliable assessment scale indicator known as **ChlorTox Scale**, regarding health and environmental risks associated with chemicals’ use. This scale is more preferred than the “Risk Assessment Matrix” method which depends on individual subjective judgement especially when selecting risks’ likelihood and severity, leading to uncertainty and inappropriate guidance for control measures [32]. ChlorTox Scale aligns with the “Greenness” definition from mathematical aspect, as proposed through UGT (Unified Green Theory). This latest approach is recommended for quantitative analytical methods’ evaluation, as well it is based mainly on estimating two variables: (h) chemical hazards and (q) quantity. The following equation signifies theoretically the calculation of ChlorTox, that is expressed in the mass of chloroform (g):

**ChlorTox = CH**_**Sub.**_**/ CH**_**CHCl3**_**. m**_**Sub.**_, where (h) is denoted as the ratio of CH_Sub_. (substance of interest) to CH_CHCl3_ (chloroform), and (q) is denoted as the m_Sub_. (mass of substance of interest). Firstly, chemical hazards (h) are calculated using Weighted Hazards Number (WHN); a danger categorization model for quick hazard assessment, WHN is calculated through the summation of hazards mentioned in MSDS (*Sect.* (Experimental); named hazards identification) of substances of interest and/or chloroform, then each hazard category is given a weight as follows: hazards of category 1 *(N*_*cat1*_*)* = 1, hazards of category 2 *(N*_*cat2*_*)* = 0.75, hazards of category 3 *(N*_*cat3*_*)* = 0.5, hazards of category 4 *(N*_*cat4*_*)* = 0.25. Consequently, WHN equation is calculated as follows: **CH**_**sub**_= 1*N_***cat1***_+ 0.75*N_***cat2***_+ 0.5*N_***cat3***_+ 0.25*N_***cat4***_, where ***N***_***cat***_ is no. of hazards per category in MSDS. Finally, for ensuring method assessment reliability, it is recommended to unify all the safety data supplier (MSDS) used in the whole process. Secondly, m_Sub_ of single analysis (q) is calculated using the equation: **m**_**Sub**_$\:=\frac{\varvec{m}+\varvec{m}\varvec{{\prime\:}}}{\varvec{N}}$, where ***m*** is the direct consumption of substance mass, ***m’*** is the consumed substance mass for extra mandatory steps, such as rinsing and calibration, and ***N*** is no. of consecutive analyses [29].

ChlorTox scale risk for ethanol was assessed and found to be 2.06 g regarding the proposed spectrophotometric methods; ensuring low hazardous solvent use (Table 6). This method is necessarily used for estimating chemical hazards only, yet other method’s details including sustainability, validity, energy, procedure, and time were not covered. Accordingly, the previous method empowered the needed assessment in aspects of greenness, safety and ecology.

**Table 6:**
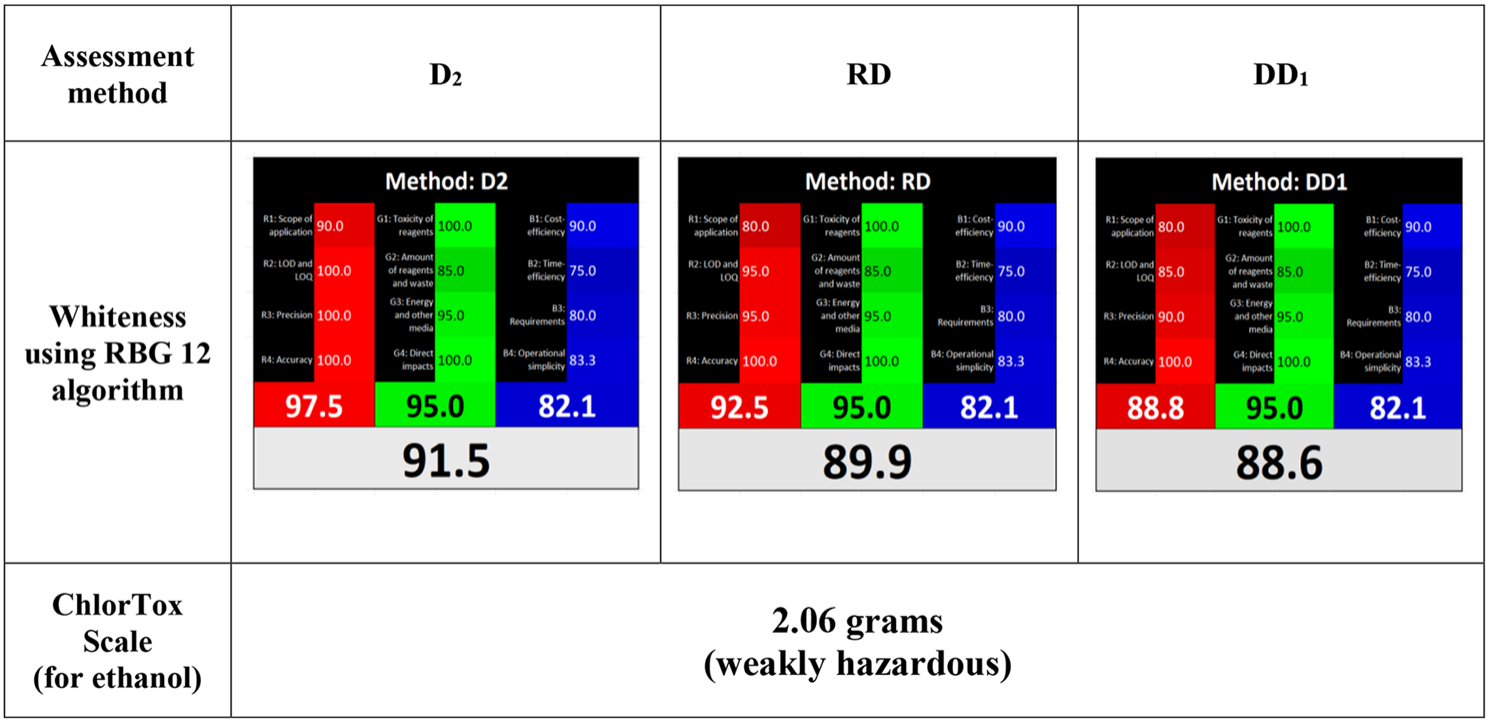
Whiteness and ChlorTox Scale assessment for the proposed spectrophotometric methods

## Conclusion

As stability-indicating studies expose significant need to be developed; for confirming drug stability and its regulatory necessity. Additionally, spectrophotometric methods-based efficiency returns mainly to its simplicity, low-cost, validity, accuracy, and wide applicability. In this work, three simple, precise, and rapid stability-indicating spectrophotometric manipulations were successfully developed for quantitative analysis of LTZ in presence of up to 90% alkali-induced degradation products. Methods’ validity were performed as per ICH guidelines, additionally, they were applied in bulk powder, laboratory-prepared mixtures, and pharmaceutical formulation. Furthermore, whiteness tool was used to evaluate the proposed methods through the RBG12 algorithm, as well, ChlorTox scale was used to assess chemicals’ hazards of ethanol.

## Electronic supplementary material

Below is the link to the electronic supplementary material.

Supplementary Material 1

## Data Availability

All data produced or analyzed during this research are provided in this published article or supplementary information.
