# Supplementary material for: Stability-indicating spectrophotometric manipulations for the determination of Letrozole in the presence of its alkali-induced degradation products; towards whiteness and ChlorTox scale perspectives
Source: BMC Chem. 2025 Mar 8;19(1):63. doi: 10.1186/s13065-025-01416-2 (PMC11890550; doi:10.1186/s13065-025-01416-2)
Supplement: Supplementary file 1 — Supplementary Material 1 [file 13065_2025_1416_MOESM1_ESM.docx]

**
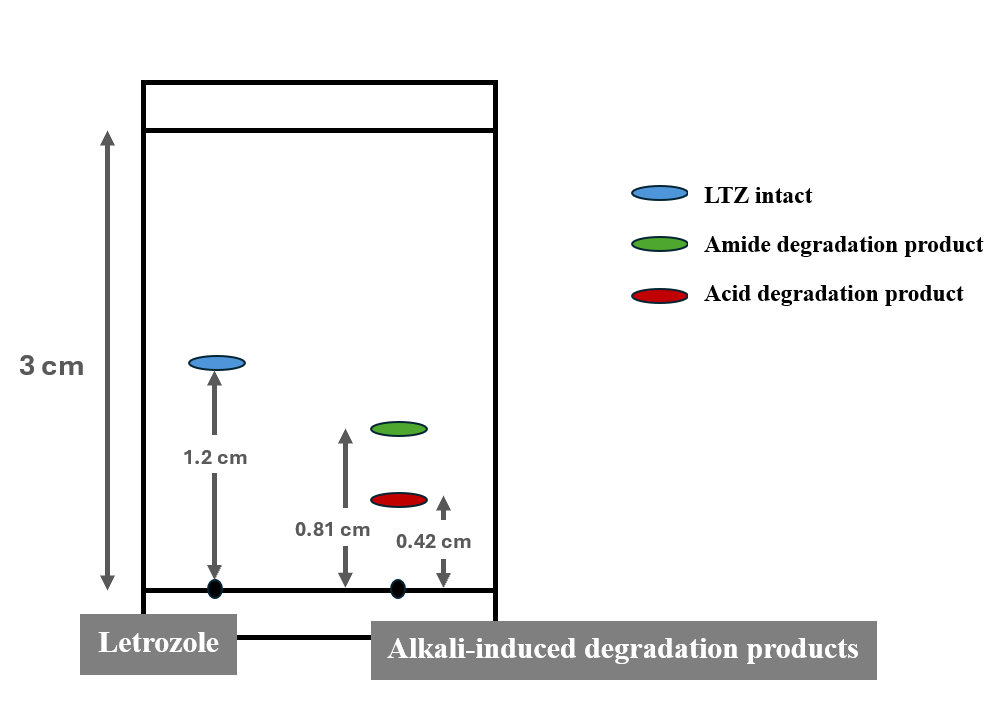
Supplementary data**

**(S1a)**


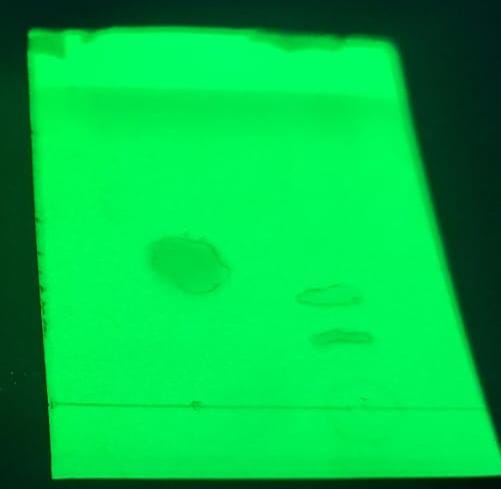


**(S1b)**

**Fig. S1.** Complete alkali-induced degradation products of Letrozole was confirmed using TLC development system composed of hexane: ethyl acetate (1:1 v/v), **(S1a)** **illustrative drawn photo** and **(S1b) real TLC photo.**

**Fig. S2.** Calibration curve for Letrozole determination alone (1.00-16.00 µg/mL).

**(S3a)**

**(S3b) (S3c)**

**Fig. S3.** Calibration curves for Letrozole determination in presence of its alkali-induced degradation products (10.00 µg/mL) **(S3a) Second derivative** (1.00-16.00 µg/mL), **(S3b) Ratio difference** (3.00-16.00 µg/mL), and **(S3c) First derivative of ratio spectra** (3.00-16.00 µg/mL).
